# Identification of MCM2-Interacting Proteins Associated with Replication Initiation Using APEX2-Based Proximity Labeling Technology

**DOI:** 10.3390/ijms26031020

**Published:** 2025-01-25

**Authors:** Sitong Yao, Zhen Yue, Shaotang Ye, Xiaohuan Liang, Yugu Li, Haiyun Gan, Jiaqi Zhou

**Affiliations:** 1 College of Veterinary Medicine, South China Agricultural University, 483 Wushan Road, Guangzhou 510642, China; sitong1129@163.com (S.Y.); yeshaotang@scau.edu.cn (S.Y.); xhliang@scau.edu.cn (X.L.); 2 Guangdong Provincial Key Laboratory of Synthetic Genomics, Key Laboratory of Quantitative Synthetic Biology, Shenzhen Institute of Synthetic Biology, Shenzhen Institute of Advanced Technology, Chinese Academy of Sciences, Shenzhen 518055, China; zhen.yue@siat.ac.cn (Z.Y.); hy.gan@siat.ac.cn (H.G.)

**Keywords:** APEX2, MCM2, DNA replication, proximity labeling

## Abstract

DNA replication is a crucial biological process that ensures the accurate transmission of genetic information, underpinning the growth, development, and reproduction of organisms. Abnormalities in DNA replication are a primary source of genomic instability and tumorigenesis. During DNA replication, the assembly of the pre-RC at the G1-G1/S transition is a crucial licensing step that ensures the successful initiation of replication. Although many pre-replication complex (pre-RC) proteins have been identified, technical limitations hinder the detection of transiently interacting proteins. The APEX system employs peroxidase-mediated rapid labeling with high catalytic efficiency, enabling protein labeling within one minute and detection of transient protein interactions. MCM2 is a key component of the eukaryotic replication initiation complex, which is essential for DNA replication. In this study, we fused MCM2 with enhanced APEX2 to perform in situ biotinylation. By combining this approach with mass spectrometry, we identified proteins proximal to the replication initiation complex in synchronized mouse ESCs and NIH/3T3. Through a comparison of the results from both cell types, we identified some candidate proteins. Interactions between MCM2 and the candidate proteins CD2BP2, VRK1, and GTSE1 were confirmed by bimolecular fluorescence complementation. This research establishes a basis for further study of the component proteins of the conserved DNA replication initiation complex and the transient regulatory network involving its proximal proteins.

## 1. Introduction

Accurate and efficient DNA replication is crucial for maintaining the stability of mammalian genomes and ensuring proper development and differentiation. This process is tightly regulated in a cell cycle-dependent manner [1]. Disruptions in DNA replication and cell cycle dynamics are associated with numerous Mendelian genetic disorders, including severe conditions such as Meier–Gorlin syndrome, natural killer cell deficiency, and familial deafness [2]. Therefore, elucidating the DNA replication process is of paramount importance. DNA replication initiates during the G1 phase of the cell cycle with the assembly of the pre-RC at numerous replication origins. As cells transition from the G1 to the S phase, the pre-RC is transformed into a pre-initiation complex (pre-IC), activating the replication helicase. This activation triggers DNA unwinding and the commencement of DNA synthesis. The process involves the sequential and interdependent recruitment of various proteins [3]. The core mechanisms and participants in DNA replication, such as ORC, CDC6, and MCM2-7, appear to be highly conserved from yeast to mammals [3,4]. Despite the identification of many protein factors involved in regulating the initiation of mammalian DNA replication, traditional analytical methods (e.g., pull-down assays, immunoprecipitation, and tandem affinity purification) predominantly capture only the most stable interaction partners [5,6]. This limitation likely results in the omission of transient and/or weak protein interactions. Consequently, while the framework of replisome assembly is understood, the dynamics of individual proteins on DNA and their contributions to the correct formation of complexes remain largely unexplored [4]. To better understand replication initiation, identifying the protein factors involved is essential.

Minichromosome maintenance (MCM) proteins are essential replication initiation factors in eukaryotes necessary for maintaining miniature chromosomes. The most well-known family within this group, MCM2-7 (minichromosome maintenance complex component 2-7), consists of six structurally related proteins which are evolutionarily conserved across all eukaryotes. The MCM2-7 proteins form a hexameric complex that serves as the replicative helicase necessary for the initiation and elongation of “once-per-cell-cycle” DNA replication in eukaryotic cells. Aberrant expression and activation of MCM2-7 directly affect DNA replication, leading to genomic instability, and are associated with tumorigenesis [7,8]. In early G1 phase, the MCM2-7 complex is a crucial component of the pre-RC assembled at replication origins [4]. During the G1-S phase transition (hereinafter referred to as the G1/S phase), the MCM2-7 proteins, along with other recruited DNA replication initiation factors, form the pre-IC [3]. The pre-IC serves as a precursor to the functional replication fork and subsequently transforms into the CMG (cell division cycle 45 (Cdc45), MCM2-7, GINS complex subunit (GINS)) helicase, which is essential for unwinding DNA during replication [4,9,10]. MCM2 acts as the core subunit of the MCM2-7 complex [11] and contains a nuclear localization signal sequence essential for the nuclear translocation of MCM family proteins [12,13], playing a pivotal role in DNA replication. Therefore, we initiated our further exploration starting with MCM2.

Identifying proteins involved in specific biological processes is crucial for understanding cellular physiology. In vivo protein–protein interaction (PPI) studies are widely used for this purpose. To overcome the limitations of traditional methods, proximity labeling techniques like APEX2 have been developed. Unlike proximity-dependent biotin identification (BioID), which requires extended labeling times [14,15], or the antibody-dependent SPLAAT and EMARS methods [16,17], APEX2 enables rapid, high-resolution labeling in a significantly shorter timeframe. When fused to a target protein and activated with biotin-phenol (BP) and hydrogen peroxide (H_2_O_2_), APEX2 generates short-lived (<1 ms) and small-radius (<20 nm) biotin-phenoxyl radicals that covalently bind to proximal proteins, allowing for their rapid labeling [6,18]. We present a method that integrates APEX2-based proximity labeling with high-resolution quantitative mass spectrometry. Targeting MCM2, we labeled proteins proximal to the replication initiation complex in mouse embryonic stem cells and fibroblasts. This approach uncovered novel interaction partners, providing new insights into the DNA replication process.

## 2. Results

### 2.1. Construction of a Functional MCM2-V5-APEX2 Fusion Protein for Proximity Labeling and Validation in Stably Transfected Cell Lines

**Figure 1:**
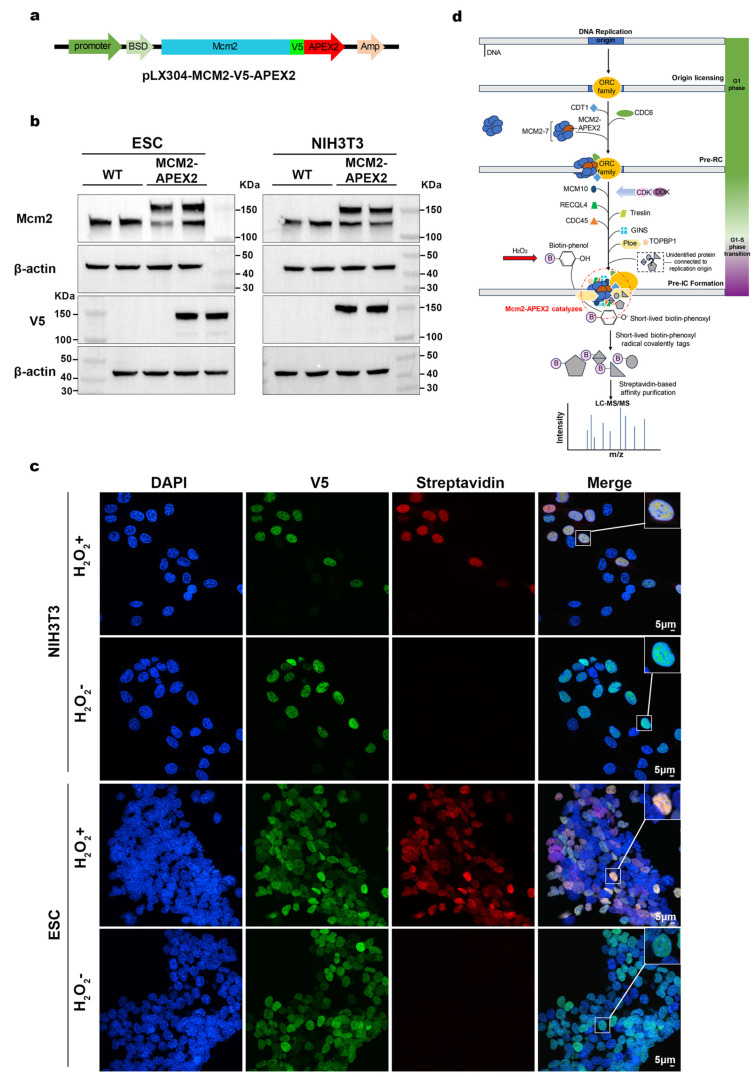
Construction of fusion protein plasmids and validation of stably transfected cell lines. (**a**) Schematic representation of the functional elements in the MCM2-V5-APEX2 fusion protein plasmid. (**b**) Validation of MCM2 protein and V5 tag expression. MCM2-APEX2: cells stably overexpressing the MCM2-V5-APEX2 fusion protein; WT: ESC and NIH/3T3 wild-type cells. (**c**) Immunofluorescence images show co-immunostaining of the DAPI (blue), V5 tag (green) and streptavidin (red) in Mcm2-APEX2 cells derived from ESC and NIH/3T3 cell lines, with or without hydrogen peroxide treatment. Localization is observed in the nucleus. The inset displays a magnified portion of the cell. DAPI: 4′,6-diamidino-2-phenylindole; scale bar: 5 μm; H_2_O_2_+: treated with hydrogen peroxide; H_2_O_2_−: untreated with hydrogen peroxide. (**d**) Diagram illustrating the formation of the DNA replication initiation complex and the APEX2 labeling process. The DNA replication initiation complex containing MCM2 is assembled during the G1-G1/S phase. At the G1/S transition, BP and H_2_O_2_ are added and incubated for 1 min to induce biotinylation of proteins within 20 nm of APEX2. LC-MS/MS: liquid chromatography–tandem mass spectrometry; pre-RC: pre-replication complex; pre-IC: pre-initiation complex. All experiments were conducted with three biological replicates.

To investigate the proteins proximal to DNA replication origins as well as to identify novel DNA replication initiation-related interacting proteins associated with the MCM2 protein within the replication initiation complex, we first constructed a fusion vector (Figure 1a). This vector included the target gene *Mcm2*, an N-terminal V5 tag, and the peroxidase APEX2, together with two different selection markers: ampicillin (Amp) and blasticidin (BSD). Amp was used for positive clone selection in E. coli, while BSD was used for selecting stably transfected cells. Subsequently, we employed a lentiviral infection method to generate stable cell lines expressing the MCM2-V5-APEX2 fusion protein in both mouse ESCs and NIH/3T3. Using Western blot analysis with a V5 antibody, a distinct band corresponding to the expected size of the MCM2 fusion protein was detected in the transfected cell lines, while no such band was observed in the wild-type cell lines. This result confirmed the successful establishment of the stable cell lines (Figure 1b). Flow cytometry analysis was conducted on cell lines stably expressing the MCM2-V5-APEX2 fusion protein. Under physiological conditions, no significant differences in the cell cycle were observed between wild-type cells and the fusion protein-expressing cell lines, indicating that MCM2 overexpression did not negatively affect cell proliferation (Figure S5).

To further verify the biological activity of the MCM2-V5-APEX2 fusion protein within these cell lines, we characterized the fusion protein and APEX2 enzyme activity via immunofluorescence. Cells were treated with H_2_O_2_ for 1 min in the presence of biotin-phenol (BP), followed by fixation and permeabilization. Antibody staining was then performed to visualize the fusion protein and biotinylation signal. Fluorescent imaging revealed that the V5 tag signal overlapped with the biotinylation signal, and both were localized within the nucleus (Figure 1c). In contrast, no biotinylation signal was observed in the negative control group (samples not treated with H_2_O_2_), which demonstrated that the fusion protein was enzymatically active and capable of labeling proteins proximal to MCM2.

### 2.2. Identification of the Proteome Proximal to MCM2

In order to ensure the accuracy of genomic DNA replication during cell division, strict regulation is implemented to guarantee that only one round occurs per cell cycle, both in terms of timing and location. As illustrated in Figure 1d, the assembly of the replication complex (RC) begins with the recruitment of the origin recognition complex (ORC) family to the origin of replication [19]. During the G1 phase, ORC family proteins are recruited to the origins, followed by the recruitment of cell division cycle 6 (CDC6) and chromatin licensing and DNA replication factor 1 (CDT1). The proper loading of CDT1 and CDC6 facilitates the recruitment of the MCM2 ring, which includes the seven subunits of MCM2-7. At this stage, the RC is referred to as the pre-RC, with the DNA helicase MCM ring fully loaded in an inactive double hexamer form [20]. During the G1/S phase, several factors are recruited to prepare for the assembly of the pre-IC. This process is driven by a series of phosphorylation events mediated by Dbf4-dependent kinases (DDKs) and cyclin-dependent kinases (CDKs). These factors include CDC45, MCM2-7, and the GINS. MCM is a key target of DDK activity, while CDK phosphorylates essential pre-initiation factors such as DNA topoisomerase 2-binding protein 1 (TOPBP1), the interaction checkpoint and replication regulator (TRESLIN), and ATP-dependent DNA helicase Q4 (RECQL4). This enables BRCT-dependent binding of TOPBP1 and the coordinated recruitment of CDC45 and GINS1-4 with DNA polymerase ε (Pol ε) [21,22,23,24,25]. These proteins collectively form the pre-IC [3]. Hence, before performing proximity labeling, the cells were synchronized at the G1/S phase through the double-thymidine block method, as described in previous articles [26,27,28,29]. This ensured maximal enrichment of the bait protein, MCM2, within the replication initiation complex. Proximity labeling was then performed, with asynchronous cells used as the control group.

**Figure 2:**
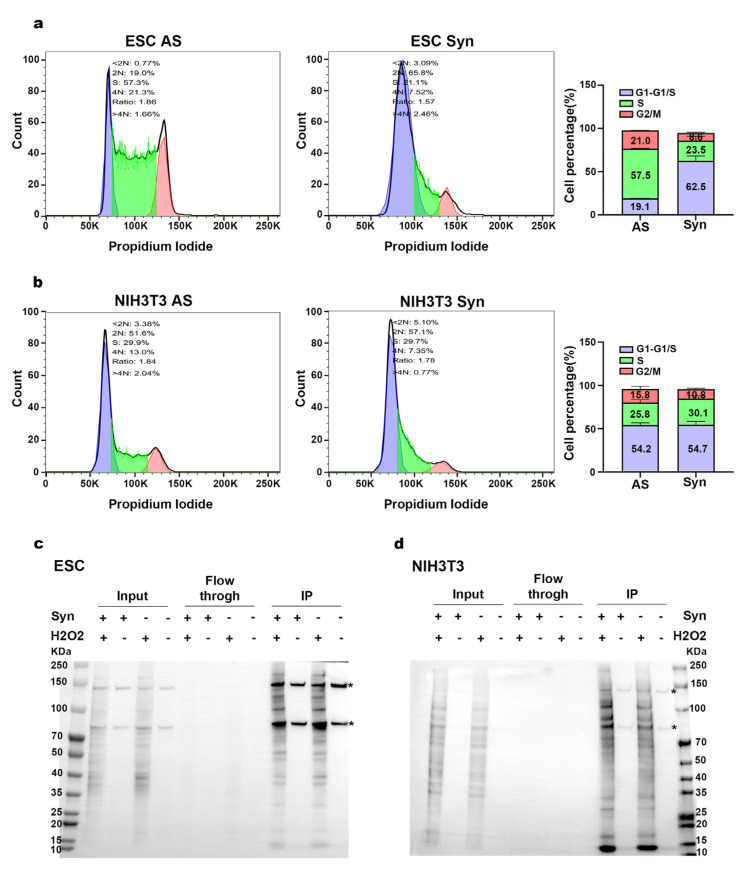
FACS analysis of cell cycle synchronization and APEX2 proximity labeling. (**a**,**b**) FACS analysis of PI-stained synchronized and unsynchronized ESC and NIH/3T3 MCM2-APEX2 cell lines. The values indicated on the bar chart represent the mean percentages of each cell cycle phase, calculated from three independent biological replicates. Detailed data can be found in Additional file S2. (**c**,**d**) Streptavidin–HRP Western blot detection of H_2_O_2_-induced protein biotinylation in cell lysates from ESC or NIH/3T3 MCM2-APEX2 cells. Asterisks denote endogenous biotinylated proteins. Syn: synchronized; AS: asynchronous.

Following synchronization, flow cytometry analysis (FACS) demonstrated that most ESC and NIH/3T3 cells were concentrated in the G1/S phase (Figure 2a,b). In ESCs, the proportion of synchronized (Syn) cells at the G1/S phase was 43.4% higher compared to asynchronous (AS) cells. Under physiological conditions, the majority of asynchronous NIH3T3 cells reside in the G1 phase. While synchronization treatment minimally affected the G1/S phase ratio, peak distribution analysis showed increased cell accumulation in the G1/S phase. To further validate the effectiveness of the synchronization protocol in NIH/3T3 cells, synchronized cells were released for 6 h post-treatment. After release, most synchronized cells progressed to the G2/M phase (%), whereas asynchronous cells remained in the G1 phase (Figure S3). Meanwhile, proximity labeling was performed, with samples not treated with H_2_O_2_ (−H_2_O_2_) serving as negative controls. After cell lysis, biotinylated proteins were enriched using streptavidin beads. Protein blotting of the input, flow-through, and bead elution fractions confirmed the success of the enrichment protocol. The findings indicated that cells treated with H_2_O_2_ generated a large number of biotinylated proteins, the majority of which were trapped and released by the magnetic beads. In contrast, cells in the negative control group produced much fewer biotinylated proteins, with hardly any biotinylated proteins present in the flow-through fractions (see Figure 2c,d). Next, the proteins captured on the beads were digested with trypsin to generate peptides for a bottom-up proteomics approach. LC-MS/MS analysis was then performed on the samples to generate proteomics data.

### 2.3. Mass Spectrometry Data Analysis

Both the ESC and NIH/3T3 cell lines treated with H_2_O_2_ were divided into synchronized (syn+) and unsynchronized (con+) groups, along with corresponding negative control groups (syn− and con−), each with two biological replicates. The raw protein MS data obtained were processed using MaxQuant (version 1.6.10.43) to generate log-transformed and mean-centered intensities of the proximal proteins. To validate the sample quality, principal component analysis (PCA) and protein abundance were used to assess the samples (Figure S4b,c). PCA demonstrated strong sample parallelism, supporting the feasibility of further analysis.

**Figure 3:**
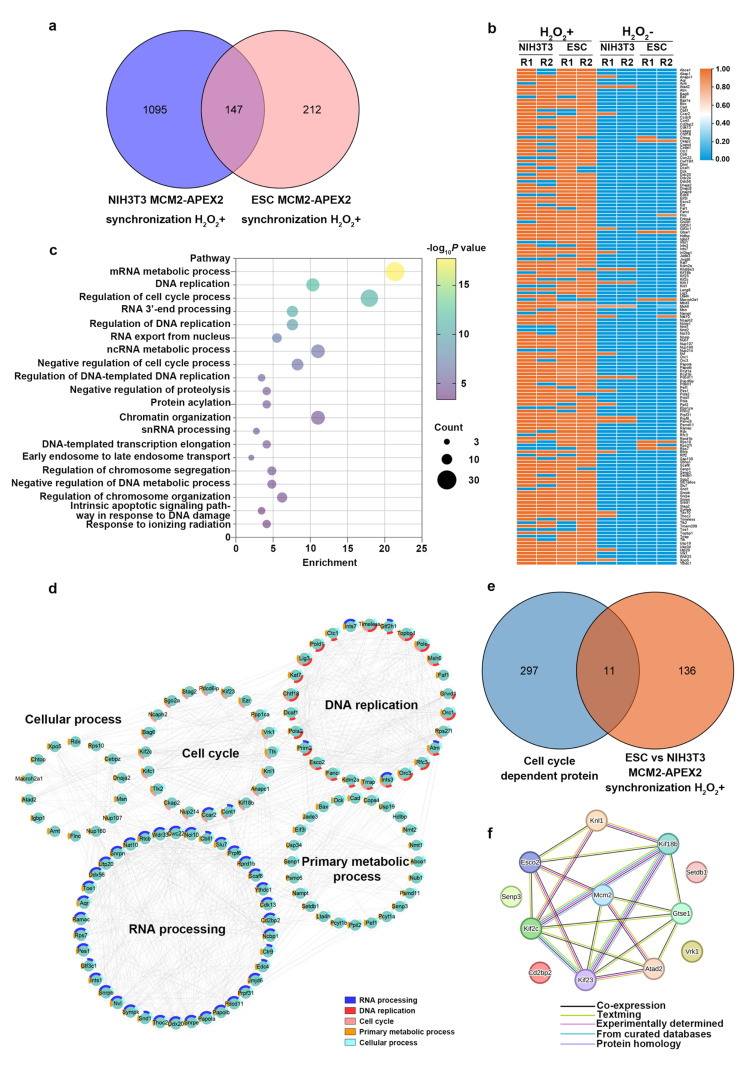
Analysis of MCM2-APEX2 proximal proteome data. (**a**) The Venn diagram illustrates the proximal proteins of the DNA replication initiation complex identified from synchronized ESC-MCM2-APEX2 and NIH/3T3-MCM2-APEX2 cells. (**b**) The heatmap shows how the expression levels of shared proteins varied among various samples. Log-transformed proximal protein data were processed using MaxQuant after the addition of H_2_O_2_ (H_2_O_2_+). The experiment without H_2_O_2_ (H_2_O_2_−) served as a control. Two biological replicates were used for each sample (R1 and R2). Expression data were normalized and presented using log_10_ transformation. (**c**) GO enrichment analysis of overlapping MCM2 proximal proteins in ESC and NIH/3T3 based on biological processes, with bubble color indicating −log_10_ *p*-values of enrichment terms. (**d**) Network visualization of selected GO biological processes associated with the MCM2 proximal proteome, with individual proteins represented as nodes and interactions as edges. Interactions were retrieved from the STRING database with a confidence score > 0.4. Node colors represent the biological processes involved. (**e**) Venn diagram comparing cell cycle-dependent proteins from the Human Protein Atlas (HPA) database with 147 overlapping proteins. (**f**) Interaction network of Mcm2 and potential interacting proteins analyzed using STRING, with interaction scores > 0.4.

To decrease false positives, proteins from the negative control groups were filtered out (log_2_ FC > 1). In the ESC cell line, synchronized data were compared with unsynchronized data. Proteins with an average quantitative abundance at least two times higher (log_2_ FC > 1) than the control group were considered specific to the synchronized group. This filtering identified 359 proteins specific to the G1/S phase (Figure 3a). Due to the high proportion of NIH/3T3 background unsynchronized cells in the G1 phase (>50%), applying the same filtering standard would exclude most proteins. Ultimately, 1242 positive proteins enriched in NIH/3T3 cells were retained (Figure 3a). Positive proteins identified in both cell lines included MCM2-7 and MCMBP, which are MCM family proteins involved in DNA replication, validating the dataset.

To identify developmentally conserved DNA replication initiation complex proximal proteins, the datasets from ESC and NIH/3T3 were intersected, resulting in 147 overlapping proteins (Figure 3a). Heatmaps showed that these 147 proteins were highly dependent on the APEX2 reaction group, with minimal protein expression in the negative control group (Figure 3b). Gene Ontology (GO) enrichment analysis of the 147 proteins revealed enrichment in Biological Process terms related to DNA replication, such as “DNA replication”, “regulation of cell cycle process”, “regulation of DNA replication”, and “regulation of DNA-templated DNA replication” (Figure 3c). Visualization of GO biological process subcategories highlighted common proteins involved in DNA replication, such as DNA ligase III (*Lig3*), *Orc1*, *Orc3*, DNA polymerase alpha 2 (*Pola2*), DNA polymerase delta 1 (*Pold1*), timeless circadian clock 1 (*Timeless*), and *Topbp1* (Figure 3d). These analyses indicated that the MCM2 proximal labeling proteome contained numerous proteins involved in DNA replication, confirming the success of our MCM2 proximal labeling strategy in identifying developmentally conserved replication initiation complex proximal proteins.

Because of the numerous potential candidates and the close connection between DNA replication and the cell cycle, we proceeded with a final enrichment step. By comparing the 147 overlapping proteins with the 307 cell cycle-dependent proteins in the Human Protein Atlas database (https://www.proteinatlas.org (accessed on 9 July 2024)) [30], we narrowed the list down to 11 proteins (Figure 3e). Subsequently, a PPI network diagram was obtained from the STRING database for MCM2 and the 11 potential interacting proteins (confidence score > 0.4; see Table 1 for a detailed list). The results showed that, except for the establishment of sister chromatid cohesion N-acetyltransferase 2 (ESCO2) and ATPase family AAA domain-containing 2 (ATAD2), for which there was experimental evidence of interaction with MCM2, the other proteins had no confirmed interaction with MCM2 (Figure 3f). Therefore, further validation experiments were conducted to identify novel replication initiation complex proximal interacting proteins.

### 2.4. Verification of Interacting Proteins

Bimolecular Fluorescence Complementation (BiFC) is a widely adopted technique for studying PPIs within living cells [31,32]. For this investigation, MCM2 was fused with the N-terminal amino acid residue 154 (N154) of the mScarlet3 fluorescent protein, while target proteins were fused with the C-terminal amino acid residue 155 (C155) of mScarlet3. The interaction between MCM2 and the target proteins brings N154 and C155 into proximity, thereby reconstituting a functional mScarlet3 fluorescent protein and enabling the visualization of the interaction.

**Figure 4:**
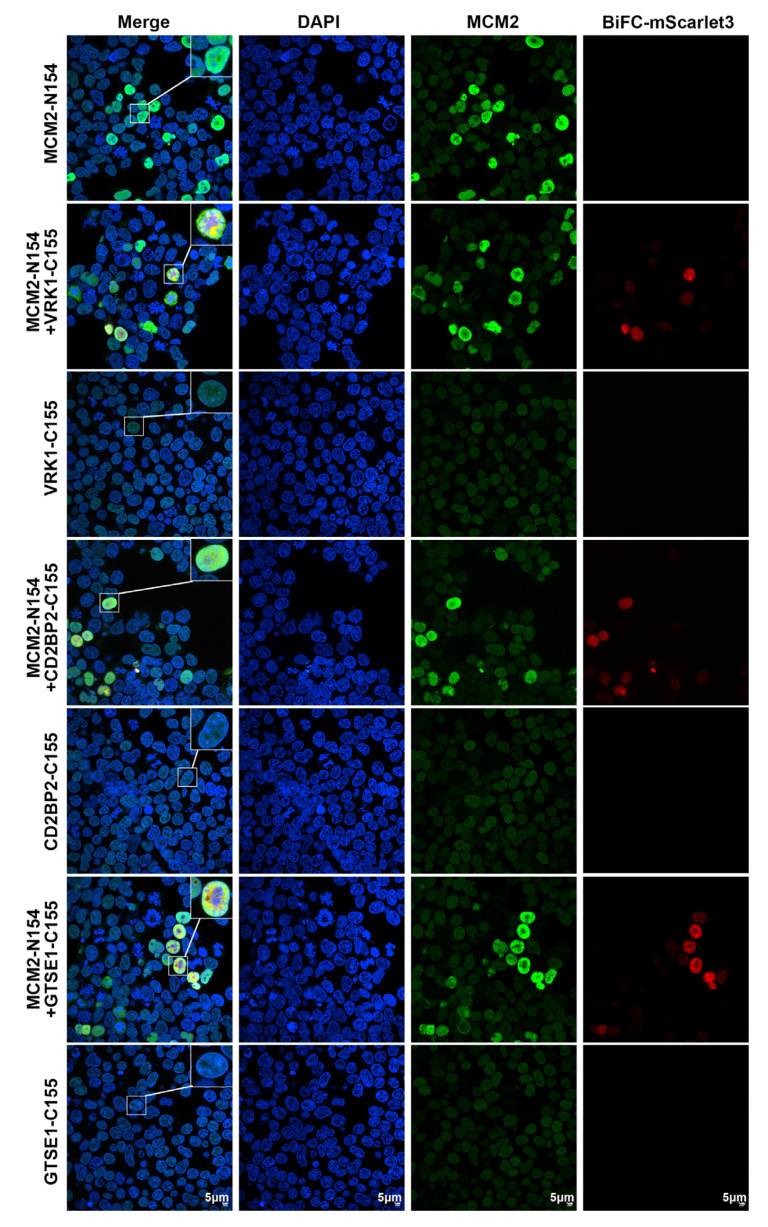
Visualization of MCM2 protein interactions with potential interacting proteins in living cells using BiFC analysis. The inset displays a magnified portion of the cell. DAPI (blue): 4′,6-diamidino-2-phenylindole; green: immunofluorescence staining with MCM2 antibody; red: BiFC-mScarlet3 fluorescent protein.

Among the proteins we examined, we selected several proteins with the least evidence of interaction for subsequent verification. C155 fusion constructs of GTSE1, VRK1, CD2BP2, SENP3, and SETDB1 were co-transfected with MCM2-N154 into HEK293T cells. The results indicated that SENP3-C155 and SETDB1-C155 did not produce fluorescence when co-transfected with MCM2-N154 (Figure S4a). In contrast, co-transfections of VRK1-C155, CD2BP2-C155, and GTSE1-C155 with MCM2-N154 resulted in the expression of fluorescence and co-localized with MCM2, as confirmed by immunofluorescence. Fluorescent protein expression was not detected when MCM2-N154, VRK1-C155, CD2BP2-C155, and GTSE1-C155 were transfected separately (Figure 4). This confirms that the observed fluorescence was not a false positive and demonstrates PPIs between MCM2 and VRK1, CD2BP2, and GTSE1.

These findings validate the use of MCM2, a critical component of the replication initiation complex, as a bait protein in combination with APEX2 peroxidase for proximity labeling. This strategy effectively identifies proteins proximal to the replication initiation complex. Further verification of the target protein requires further follow-up experiments.

## 3. Discussion

Accurate regulation of DNA replication is crucial for the faithful division of cells, which is essential for the continuation of life. The disruption of DNA replication caused by stress is a primary source of genomic instability, resulting in human genetic diseases. The regulation of proteins during DNA replication in vertebrate cells is critically important. For instance, the absence of human DNA replication-associated topoisomerases has been shown to lead to genomic instability [33], while deficiencies in functional Fanconi anemia proteins result in replication fork stalling and DNA damage [34]. Moreover, rare human disorders like Meier–Gorlin syndrome have been linked to mutations in DNA replication initiation factors such as the ORC family, CDT1, CDC6, CDC45, MCM5, and the recently discovered DNA replication fork stabilization factor DONSON [35,36]. Mutations in MCM4, GINS, and replication initiation factors (MCM10) have been linked to natural killer cell deficiencies [2,37,38,39], and a missense mutation in MCM2 has been associated with neurogenic hearing loss [40]. Hence, it is essential to ascertain the functions performed by various proteins participating in the DNA replication process. Even though numerous relevant functional proteins have been identified to date, there is still a necessity to delve deeper into the interactions among known or unknown proteins to gain a more thorough understanding of the biological process of DNA replication.

Traditional methods for studying PPIs have relied heavily on co-immunoprecipitation (co-IP) and its derivatives, such as pull-down assays or tandem affinity purification [41]. These techniques use antibodies to enrich target proteins (POIs) and their strong binding partners from cell lysates [42]. Despite its transformative utility, co-IP has well-known limitations, including difficulty in detecting transient and weak interactions, particularly when harsh conditions are required for cell lysis and protein solubilization [43]. Additionally, the dependency on effective and selective antibodies, which are not always available, further hampers this approach. Recently, proximity-dependent labeling methods have been developed as novel tools for identifying PPIs. These include the BioID method [44,45], TurboID [46], and the pupylation-based PUP-IT method for membrane protein interactions [47]. Each system has its limitations. BioID requires 15–18 h for labeling. Despite the fact that TurboID and miniTurbo can speed up the process to less than 10 min, prolonged biotin incubation can potentially interfere with cellular protein functions, induce artificial interactions, result in cytotoxicity, and hinder the accurate capture of PPIs at specific time points [46,48]. Additionally, PUP-IT may not be suitable for organelle interactions, as Pup, a 64-amino acid prokaryotic protein, cannot diffuse across membranes [49]. The APEX2 proximity labeling method was selected for its reduced toxicity and quicker reaction times in this study. When activated by H_2_O_2_, APEX2 produces biotin-phenol radicals, allowing for protein labeling in under a minute. By rapidly labeling proteins and synchronizing the cell cycle, we can accurately capture interacting proteins at the exact moment of replication initiation.

MCM proteins are essential for DNA replication initiation and critical for maintaining genomic stability [50,51]. These proteins are indispensable for cell growth and DNA replication [52,53]. Homologs of MCM2-7 have been identified across all eukaryotes, from yeast to humans. Phylogenetic analysis of eukaryotic MCM sequences indicates that MCM sequences are highly conserved, with 450–600 amino acids conserved within each class [4]. Although various MCM homologs and mutants have been identified in different organisms, these discoveries are often based on non-replication-related phenotypes [54,55,56], underscoring the evolutionary conservation of MCMs in DNA replication. The conservation of MCM proteins in DNA replication is preserved across different mammalian cells. Given this conservation, two cell types at different developmental stages were used in this study to investigate novel interacting proteins involved in the DNA replication process in mammals.

MCM2, an essential component of the MCM complex, plays a crucial role in DNA replication. Abnormal phosphorylation of MCM2 has been associated with replication issues [57,58,59]. Hydroxyurea (HU) induces hyperphosphorylation of MCM2 at Ser40/53/108, hindering its chromatin dissociation and disrupting DNA replication [59]. Under replication stress, PTEN regulates MCM2 to maintain genomic stability [60]. MCM2 dysfunction, by affecting DNA replication and cell proliferation, contributes to the development of cancer [8,61,62]. Hence, MCM2 was chosen as the target protein for this research to endogenously label the proximal proteins of the DNA replication initiation complex. Despite using an overexpression system, the “MCM paradox” [10,63] justifies this approach, as excess MCM can act as “dormant origins”, which is crucial for rescuing stalled replication forks and preserving genomic integrity [64]. Recent evidence suggests that MCM overabundance may slow DNA replication while enhancing replication robustness. This may explain why even slight reductions in MCM levels destabilize the genome and increase tumorigenesis [8,65]. In mammalian cells, acute MCM2 depletion has been reported to result in genomic instability and impaired cell proliferation, with MCM2 knockout leading to cell death [66,67]. Furthermore, in murine models, MCM2 reduction or loss has been associated with a range of phenotypic outcomes, including embryonic lethality, growth retardation, reduced cell proliferation, genomic instability, and early-onset tumorigenesis [68,69]. Consistent with these findings, our experiments demonstrated that MCM2 overexpression does not induce detectable physiological alterations under normal cellular conditions, thereby validating the appropriateness of this system for the present study.

GTSE1, also known as B99, is a protein-coding gene located on chromosome 22q13.2-q13.3 [70]. Studies have shown that GTSE1 expression increases significantly during the DNA synthesis phase of the cell cycle, suggesting its role in key processes like mitosis and DNA replication [71,72,73]. In human cancer cells, the absence of GTSE1 has been shown to inhibit cell proliferation. GTSE1 collaborates with other DNA replication proteins, including CDC20, PCNA, and MCM6, to regulate the cell cycle. Knockdown of GTSE1 disrupts cell cycle progression and reduces the number of EdU-positive cells, and overexpression of GTSE1 increases the number of EdU-positive cells. These findings highlight the critical role of GTSE1 in cell cycle transition and DNA replication [74,75]. GTSE1 has been identified as a negative regulator of tumor protein p53 (P53) by physically interacting with its C-terminal domain, facilitating P53′s nuclear export and degradation [72,76,77]. Cyclin-dependent kinase inhibitor 1A (P21), a crucial downstream target of P53, is a potent CDK inhibitor that binds and inactivates cyclin–CDK complexes [78,79,80]. CDK not only triggers pre-initiation complex assembly with DDK at the G1-S transition but also prevents MCM2-7 reloading during the S and G2/M phases, thereby inhibiting origin reactivation and re-replication [81,82]. It is hypothesized that nuclear GTSE1 interacts with Mcm2 in the replication initiation complex. This interaction may facilitate the downregulation of nuclear P53 and its downstream effector P21 to modulate CDK activity and prevent re-replication. Further validation of this mechanism is required.

VRK1 is a serine–threonine kinase localized in the nucleus, specifically on the chromatin of quiescent cells [83]. It forms stable complexes with various chromatin-associated proteins, including histones, transcription factors, and proteins involved in DNA repair processes. VRK1 exhibits elevated expression levels in tumor cells and normal cells and in tissues with high proliferation rates [84,85]. Depletion of VRK1 results in significant defects in cell proliferation [86,87], highlighting its essential role in cell cycle progression. Specifically, VRK1 expression peaks during the S phase of the cell cycle [88]. Overexpression of human VRK1 has been shown to enhance bromodeoxyuridine (BrdU) incorporation into DNA, indicating an increase in DNA synthesis [89]. In murine models, partial knockdown of VRK1 disrupts gametogenesis, impairing the development of spermatogonia and oocytes and ultimately leading to infertility in both male and female mice [90,91,92,93]. Furthermore, knockdown of VRK1 reduced viral replication and expression in vaccinia virus- and hepatitis C-infected cells, indicating that VRK1 protein is required for viral DNA replication [94,95]. Previous studies have revealed that covalent modifications of histones and DNA regulate all DNA-dependent processes, such as transcription, DNA repair, and replication [96]. Studies indicate that histone acetylation modulates the initiation of DNA replication and plays a critical role in the formation of the pre-RC [97]. Efficient DNA replication requires multiple acetylations on the histone H3 and H4 tails, and these acetylations are critical for activating replication origins [98]. The loading of the MCM2-7 helicase during DNA replication is also regulated by H3 and H4 acetylation, with excessive H4 acetylation leading to excessive loading of MCM2-7. Mutations that impair phosphorylation of histone acetyltransferase reduce H4 acetylation, decrease loading of Mcm2-7, and negatively impact DNA replication and cell proliferation [97]. Recent research has identified VRK1 as a candidate gene involved in the regulation of epigenetic modifications. In response to doxorubicin or ionizing radiation, VRK1 directly interacts with and phosphorylates histone H3 at the Thr3 site [99,100]. Loss of VRK1 results in reduced H3 and H4 acetylation, both under basal conditions and following DNA damage [101]. Our findings suggest that VRK1 interacts with MCM2, potentially regulating the acetylation levels of histones H3 and H4 during DNA replication. This regulation may influence the loading of MCM2-7 onto DNA, coordinating the assembly of the replication initiation complex and thereby maintaining genome stability.

CD2BP2 was initially identified as a binding partner of the adhesion molecule CD2. It functions as a pre-spliceosome assembly factor by co-localizing with spliceosome proteins via its glycine–tyrosine–phenylalanine (GYF) domain. Depletion of CD2BP2 in murine T cells has been reported to induce apoptosis and suppress cellular proliferation [102]. Moreover, conditional knockout of CD2BP2 in mice results in marked phenotypic abnormalities, including growth retardation, impaired vascular development, and embryonic lethality attributed to non-placental deficiencies [103]. These observations implicate CD2BP2 as a likely critical regulator in the initiation of DNA replication. Notably, PP1 has been identified as a novel GYF domain-independent binding partner of CD2BP2, with their interaction mediated by an N-terminal recognition motif characteristic of typical PP1 binding partners [103]. DDK-dependent phosphorylation of MCM proteins is essential for DNA replication [104,105]; however, excessive phosphorylation can disrupt the replication process [57,59]. PP1, mediated by Rif1, can reverse DDK-dependent phosphorylation across all MCM substrates. In the absence of Rif1, PP1 activity is compromised, resulting in hyperphosphorylation of MCM proteins, accelerated replication initiation, and disruption of normal replication timing. This also impairs the ability to prevent initiation under replication stress [104]. Through proximity labeling, we identified an interaction between CD2BP2 and MCM2 within the replication initiation complex, suggesting that CD2BP2 may recruit PP1 to prevent hyperphosphorylation of MCM2-7. This regulation may control origin selection and subsequent assembly of the replication initiation complex. Further experimental validation is required to confirm this hypothesis.

## 4. Materials and Methods

### 4.1. Cell Culture

Mouse E14TG2a embryonic stem cells (ESCs) (kindly provided by B. Zhu’s lab at the Institute of Biophysics, Chinese Academy of Sciences [68]) were cultured in DMEM/F-12 (Gibco, Waltham, MA, USA) coated with gelatin, supplemented with 15% (*v*/*v*) fetal bovine serum (Gibco), 1% penicillin–streptomycin (Gibco, Waltham, MA, USA), 1 mM sodium pyruvate (Gibco, Waltham, MA, USA), 2 mM GlutaMAX (Gibco, Waltham, MA, USA), 1% MEM non-essential amino acids (Gibco, Waltham, MA, USA), 55 μM β-mercaptoethanol (Sigma-Aldrich, St. Louis, MO, USA), and 10 ng/mL mouse leukemia inhibitory factor (Millipore, Burlington, VT, USA) at 37 °C in a humidified atmosphere of 5% CO_2_. Cells were passaged using trypsin–EDTA (Gibco, Waltham, MA, USA) and regularly checked for mycoplasma contamination. The mouse ESCs were validated by transcriptome analysis, showing high similarity to the E14TG2a dataset published in ENCODE (GSE66582). Mouse fibroblast cells (NIH/3T3 cells, purchased from the American Type Culture Collection, Manassas, VA, USA) were cultured in high-glucose DMEM with 10% fetal bovine serum, 100 U/mL penicillin, and 100 mg/mL streptomycin at 37 °C in 5% CO_2_. Mycoplasma contamination was tested before the experiments.

### 4.2. Construction of Plasmid Expression Vectors

The plasmid pLX304-Flag-APEX2-NES (Plasmid No. 92158) was obtained from addgene, while the *Mcm2* gene was amplified using mouse cDNA as a template. The *Mcm2* gene fragment was then recombined into the linearized pLX304-Flag-APEX2-NES using the NovoRec^®^ plus One step PCR Cloning Kit (NR005; Novoprotein, Shanghai, China). Positive clones were identified through transformation, and the resulting plasmid was named pLX304-MCM2-V5-APEX2-Mito.

### 4.3. Construction of the Mcm2-APEX2 Cell Line

The lentiviral packaging system plasmids PSPAXZ, PMD2.G, and pLX304-MCM2-V5-APEX2 were transfected into HEK293T cells. The cell culture supernatant was collected and filtered through a 0.45 μm filter membrane. The filtered supernatant was then concentrated by centrifugation. The concentrated virus solution was mixed with fresh culture medium and added to cells in the logarithmic growth phase. After 24–48 h of infection, the culture medium was replaced with BSD (10 μg/mL) for selection of resistant cells. After 5–7 days of selection, protein blotting was performed for verification. The cell line that was stably transfected with the pLX304-MCM2-V5-APEX2 plasmid was named the Mcm2-APEX2 cell line.

### 4.4. Preparation of Cell Extracts and Western Blot Analysis

The cells were lysed using RIPA buffer (Cell Signaling Technology, Cat. No. 9806, Danvers, MA, USA) and then boiled for 5 min after adding SDS loading buffer. For Western blot analysis, the following antibodies were used: MCM2 (Cell Signaling Technology, Cat. No. 3619), β-Actin (Beyotime, Cat. No. AF0003, Haimen, China), and V5-Tag (Cell Signaling Technology, Cat. No. D3H8Q, Danvers, MA, USA), all diluted to 1:1000 in 3% BSA. HRP-conjugated goat anti-mouse (Beyotime, Cat. No. A0216, Haimen, China) or HRP-conjugated goat anti-rabbit (Beyotime, Cat. No. A0208, Haimen, China) antibodies were used at a dilution of 1:5000, depending on the primary antibody source. The bands were visualized using Tanon App for Biology Software (version 1.0.0000) after incubation with ECL cocktail (Beyotime, Cat. No. P0018FM, Haimen, China). For full scan blots, please refer to Figures S1 and S2.

### 4.5. Flow Cytometry Was Performed After Cell Synchronization with Double Thymidine

When the cells reached 60% to 70% confluence, 2 mM thymidine (Sigma, Cat. No. T1895, Raleigh, NC, USA) was added and cultured for 16 h. The control group was cultured with only full nutrient growth medium. After 16 h, the cell culture was washed twice with PBS to remove any residue and replaced with full nutrient growth medium for 7 h. Following further culturing in medium containing 2 mM thymidine for 16 h, some cell samples were collected to confirm the cell synchronization effect. The cells were fixed with 75% ethanol at 4 °C overnight. After being washed twice with 1× PBS, the cells were suspended in a solution containing 50 μg/mL of propidium iodide (Sigma, P4170, Raleigh, NC, USA) and 100 μg/mL of RNase A (Sigma, R5503, Raleigh, NC, USA) and incubated at room temperature in the dark for 30 min. The stained cells were then analyzed using a flow cytometer and cell sorting workstation (BD FACSArialIII, Franklin Lakes, NJ, USA). The cell cycle distribution was examined using FlowJo version 10.8.1.

### 4.6. In Situ Labeling of MCM2 Interactors Mediated by APEX2-Mediated Biotinylation

Following two washes with medium, both synchronized and unsynchronized cells were incubated in 1 mL of medium containing 100 μM biotin-phenol for 30 min in a 37 °C incubator. The APEX2 labeling reaction was then initiated by adding H_2_O_2_ to each sample to a final concentration of 1 mM and gently mixing by tapping the tube several times. After 1 min of reaction, the reaction was halted with an equal volume of 2× quenching buffer (10 mM Trolox, 20 mM sodium ascorbate, and 20 mM sodium azide in PBS). Negative controls consisted of samples without the addition of the H_2_O_2_ solution.

### 4.7. Immunofluorescence

Cells were either treated with hydrogen peroxide (H_2_O_2_+) or left untreated (H_2_O_2_−) and then fixed with 4% paraformaldehyde in PBS for 15 min at room temperature. After three washes with PBS, the cells were blocked with 3% BSA in 0.1% PBST (blocking buffer) for 1 h at room temperature. Following this, the cells were incubated overnight at 4 °C with the primary antibody V5-Tag (1:100; Cell Signaling Technology, Cat. No. D3H8Q, Danvers, MA, USA) in blocking buffer. After three washes with PBST, the cells were incubated with the secondary antibodies Donkey anti-rabbit IgG H&L (1:200; Invitrogen, Cat. No. A32790, Waltham, MA, USA), Goat anti-rabbit IgG H&L (1:300; Invitrogen, Cat. No. 35561, Waltham, MA, USA), Alexa Fluor 594 streptavidin conjugate (1:300; Invitrogen, Cat. No. S11227, Waltham, MA, USA), and Alexa Fluor 488 streptavidin conjugate (1:300; Invitrogen, Cat. No. S11223, Waltham, MA, USA) in blocking buffer for 1 h at room temperature. The cells were then washed with Hoechst and incubated for 10 min at room temperature, followed by three washes with PBS before imaging. Images were acquired and examined using the A1R confocal microscope (Nikon, Minato-ku, Tokyo, Japan).

### 4.8. Streptavidin Pull-Down and Western Blot Analysis of Biotinylated Proteins

Cell pellets labeled with APEX2 were lysed on ice for 15 min using RIPA lysis buffer (50 mM Tris-HCl (pH 7.5), 150 mM NaCl, 1.5 mM MgCl_2_, 1 mM EGTA, 1% SDS, 1% NP-40, 0.4% sodium deoxycholate, 1 mM DTT, 1 mM PMSF, and 1× Roche Complete EDTA-free protease inhibitor tablet). The lysates were then sonicated for 3 min (100 W; 3 s on, 3 s off) and boiled at 100 °C for 10 min. After centrifugation to clarify the cell extracts, the protein content in each supernatant was quantified. Afterwards, 5% of the supernatant was set aside for Western blot analysis. The SDS in the samples was then diluted to 0.2% using 1× cold RIPA buffer (RIPA buffer that does not contain SDS). Streptavidin–sepharose beads (GE Healthcare, Shanghai, China; Cat. No. 17-5113-01, Chicago, IL, USA) were washed twice with 1× cold RIPA buffer (0.2% SDS), and each sample was incubated with 800 μg of protein and 50 μL of bead slurry for 4 h at 4 °C with rotation. Five percent of the flow-through was then retained for Western blot analysis. The beads were then washed twice with 1 ml of wash buffer (50 mM Tris-HCl (pH 7.5) and 1% SDS), twice with 1 mL of RIPA wash buffer (50 mM Tris-HCl (pH 7.5), 150 mM NaCl, 1.5 mM MgCl_2_, 1 mM EGTA, 0.2% SDS, 1% NP-40, and 1 mM DTT), twice with 1 mL of 8 M urea buffer, twice with 1 mL of 30% acetonitrile (ACN), and twice with 1 mL of 20 mM ammonium bicarbonate. Then, 5% of the beads were retained for Western blot analysis, and the remaining beads were used for LC-MS/MS analysis. For Western blot analysis, the beads were boiled in 10 μL of 5× protein loading buffer to elute the biotinylated proteins and separated by 10% SDS-PAGE. Proteins were transferred to 0.22 μm PVDF membranes, and the blots were blocked with 1% BSA in TBST for 1 h at room temperature. They were then stained with streptavidin–HRP (1:5000; Bio-Tech, Shanghai, China; Cat. No. A0303) in TBST for 1 h at room temperature. The blots underwent three 5 min washes with TBST buffer before being developed with Clarity Western ECL substrate (Bio-Rad, Cat. No. 1705060, Hercules, CA, USA) and imaged using the ChemiDoc MP imaging system by Bio-Rad.

### 4.9. On-Bead Digestion and LC–MS/MS

MS-based proteomics experiments were conducted following previously established protocols with slight adjustments [106]. In brief, the beads were enriched, washed, and then resuspended in 200 µL of on-bead digestion buffer (50 mM HEPES (pH 8.0), 1 μM CaCl_2_, and 2% ACN). Subsequently, 10 mM tris(2-carboxyethyl) phosphine (TECP; ThermoFisher Scientific, Waltham, MA, USA; Cat. No. 77720) and 40 mM chloroacetamide (CAA; Sigma, Cat. No. 194921, Raleigh, NC, USA) were added, followed by a 30 min incubation at room temperature. The beads were washed with 1 mL of on-bead digestion buffer, followed by resuspension in 100 µL of the same buffer containing endopeptidase (Wako Pure Chemical Industries, Ltd., Osaka, Japan; Cat. No. 125-05061) and incubation at 37 °C for 3 h. Subsequently, on-bead digestion buffer (Promega, Madison, WI, USA; Cat. No. V5280) with 0.5 µg of trypsin was added, and digestion was carried out at 37 °C for 16 h. Prior to LC-MS/MS analysis, samples were desalted using StageTips made of C18 material inserted into 200 μL pipette tips. Peptide samples were desalted by washing the C18 material once with 200 μL of ACN, once with 200 μL of StageTips buffer B [50% (*v*/*v*) ACN/0.1% (*v*/*v*) formic acid (FA) in H_2_O], and twice with 100 μL of StageTips buffer A [0.1% (*v*/*v*) FA in H_2_O]. After loading the peptide samples onto the StageTips and washing them twice with 100 μL of StageTips buffer A, the peptides were eluted with 100 μL of StageTips buffer C [0.1% (*v*/*v*) FA in 40% (*v*/*v*) ACN/H_2_O] and 100 μL of StageTips buffer B. The eluted fractions were collected by centrifugation at 500× *g* for 5 min at room temperature, and the solution from the peptide samples was evaporated using a SpeedVac at 45 °C. Finally, 10 μL of StageTips buffer A was added to the sample for LC-MS/MS analysis.

### 4.10. LC-MS/MS Analysis

Peptides were reconstituted in water containing 0.1% formic acid and then separated on reversed-phase columns (trap column: particle size = 3 μm, C18, length = 20 mm; analytical column: particle size = 2 μm, C18, length = 150 mm) utilizing an Ultimate 3000 RSLCnano system (ThermoFisher Scientific, Waltham, MA, USA) linked to an Orbitrap Q-Exactive HF. The separation of peptides was achieved with a 60 min gradient (buffer A: 0.1% formic acid in water; buffer B: 0.1% formic acid in 80% acetonitrile–water) at a flow rate of 300 mL/min and analyzed using the Orbitrap Q-Exactive HF in data-dependent mode. In positive-ion mode, the Orbitrap Q-Exactive HF mass spectrometer was operated with the ion-transfer tube temperature set at 275 °C. A positive-ion spray voltage of 2.1 kV was applied during data acquisition. Full-scan MS spectra ranging from *m*/*z* 350 to 2000 were recorded at a resolution of 60,000 in the Orbitrap. Higher collision dissociation fragmentation was carried out at a normalized collision energy of 28%. The MS2 automatic gain control (AGC) target was set to 5 × 10^4^ with a maximum injection time of 50 ms and a dynamic exclusion period of 30 s.

### 4.11. MS Data Analysis

The raw data for protein identification and label-free protein quantification were processed using MaxQuant (version 1.6.10.43) and its integrated Andromeda search engine for feature extraction, peptide identification, and protein inference. Peptides and proteins were identified by searching the mouse reference proteome in the UniProt database (UniProtKB/Swiss-Prot and UniProtKB/TrEMBL, version 2020_12), in addition to manually annotated contaminants. The false discovery rate (FDR) was established at 0.01, and the inter-run matching algorithm was activated. Subsequent to the search, reverse matches, contaminants, and proteins identified by only one site were eradicated (see the Additional File S1 for specific information on these proteins). The screening results were exported and subsequently visualized through the use of Python (version 3.8.3), the online gene annotation and analysis tool Metascape (http://metascape.org/gp/index.html#/main/step1, accessed on 9 June 2024), and the complex network visualization platform Cytoscape (version 3.8.2).

The transformed protein intensity of the APEX2 H_2_O_2_-added experiment compared to the no-H_2_O_2_ control experiment was calculated and expressed as a fold change (log_2_ FC). Proteins with a log_2_ FC > 1 were ultimately distinguished from background proteins in two separate measurements and were deemed non-false-positive proteins.

Gene Ontology (GO) analysis is a commonly utilized and powerful gene annotation method that can help uncover the biological characteristics of extensive genomic or transcriptomic data [107]. Metascape, an online bioinformatics tool for annotation visualization and an integrated discovery database, was employed for the analysis. The tool was employed to interpret GO functions and visually represent 147 overlapping protein-related biological processes. The graph displayed the top 20 significantly enriched GO biological process terms [108].

To visualize non-redundant biological terms in the functional grouping network, we used the Cytoscape plugin String Enrichment (version 2.1.1). The correlation between terms is illustrated by the similarity of relevant genes, with node colors switching between functional groups and cluster distributions in the network. In order to decrease redundancy, similar terms with related genes are merged [109].

The STRING database was utilized to assess the confidence of interaction between the identified proteins, with a confidence score of 0.4 and an FDR stringency of 0.05. A link was established based on this analysis. Subsequently, the PPIs of 11 potential MCM2-interacting proteins were investigated using the STRING database.

### 4.12. Bimolecular Fluorescence Complementation (BiFC)

The coding sequences of Lifeact, the N- and C-terminals of mScarlet3, were amplified by PCR from plasmid pcDNA3.1(+) Lifeact-mScarlet3-HA. The coding sequences of *MCM2*; vaccinia-related kinase 1 (*VRK1*); CD2 cytoplasmic tail-binding protein 2 (*CD2BP2*); SET domain, bifurcated 1 (*SETDB1*); SUMO/sentrin-specific peptidase 3 (*SENP3*); and G and S phase-expressed protein 1 (*GTSE1*) were amplified by PCR from cDNA of HEK293T cells. The primers for amplifying the above sequences were designed to contain 15 to 20 bp homology arms for subsequent recombination. All amplified fragments were ligated into the pcDNA3.1(+) mammalian expression vector by the NovoRec^®^ plus One step PCR Cloning Kit (NR005; Novoprotein, Shanghai, China). HEK293T cells were maintained in Dulbecco’s modified Eagle’s medium (DMEM; Procell, Wuhan, China) containing 10% fetal bovine serum (FBS; Excell Bio, Shanghai, China), supplemented with 100 U/mL penicillin, 100 mg/mL streptomycin, and 2 mM L-glutamine, at 37 °C under 5% CO_2_. HEK293T cells were routinely tested for mycoplasma contamination and were mycoplasma-negative. The day before transfection, cells were inoculated in 24-well plates. When the cells reached 70–80% confluency, each of the plasmids (1.0 μg total DNA per well) was transfected into cells using Lipofectamine 3000 (Invitrogen, Cat. No. L3000008). Cells were harvested 16 to 24 h after transfection and were imaged after co-staining with an Mcm2 antibody [Anti-MCM2 (1:100; Cell Signaling Technology, Cat. No. 3619)], as described above for immunofluorescence.

## 5. Conclusions

In this research, APEX2 proximity labeling was employed alongside mass spectrometry to identify 147 developmentally conserved proteins proximal to the replication initiation complex. Upon further comparison with 307 cell cycle-dependent proteins, 11 potential Mcm2 interactors were identified. Among them, CD2BP2, VRK1, SENP3, SETDB1, and GTSE1 were selected for BiFC validation. New interactions were confirmed between MCM2 and CD2BP2, VRK1, and GTSE1, demonstrating the utility of APEX2 proximity labeling in identifying novel components of the DNA replication initiation complex. This approach offers new methods and insights for further exploration of mammalian DNA replication processes.

## Figures and Tables

**Table 1:** List of the potential interacting proteins.

Name	Abbreviation
G2 and S phase-expressed protein 1	GTSE1
Vaccinia-related kinase 1	VRK1
Establishment of sister chromatid cohesion N-acetyltransferase 2	ESCO2
Kinetochore scaffold 1	KNL1
CD2 cytoplasmic tail-binding protein 2	CD2BP2
SET domain, bifurcated 1	SETDB1
Kinesin family member 18B	KIF18B
Kinesin family member 23	KIF23
SUMO/sentrin-specific peptidase 3	SENP3
Kinesin family member 2C	KIF2C
ATPase family, AAA domain-containing 2	ATAD2

## Data Availability

The label-free protein profiling data in this paper are stored in the ProteomeXchange Consortium [110] via the PRIDE Inspector Toolsuite [111] with the accession number PXD054939. The data access connection is http://proteomecentral.proteomexchange.org/cgi/GetDataset?ID=PXD054939 (accessed on 25 September 2024). All other data and materials used to support the findings of this study are available from the corresponding author upon request.

## Supplementary Materials

The supporting information can be downloaded at: https://www.mdpi.com/article/10.3390/ijms26031020/s1.
