# Supplementary figures and images for: Identification of MCM2-Interacting Proteins Associated with Replication Initiation Using APEX2-Based Proximity Labeling Technology
Source: Int J Mol Sci. 2025 Jan 25;26(3):1020. doi: 10.3390/ijms26031020 (PMC11816892; doi:10.3390/ijms26031020)

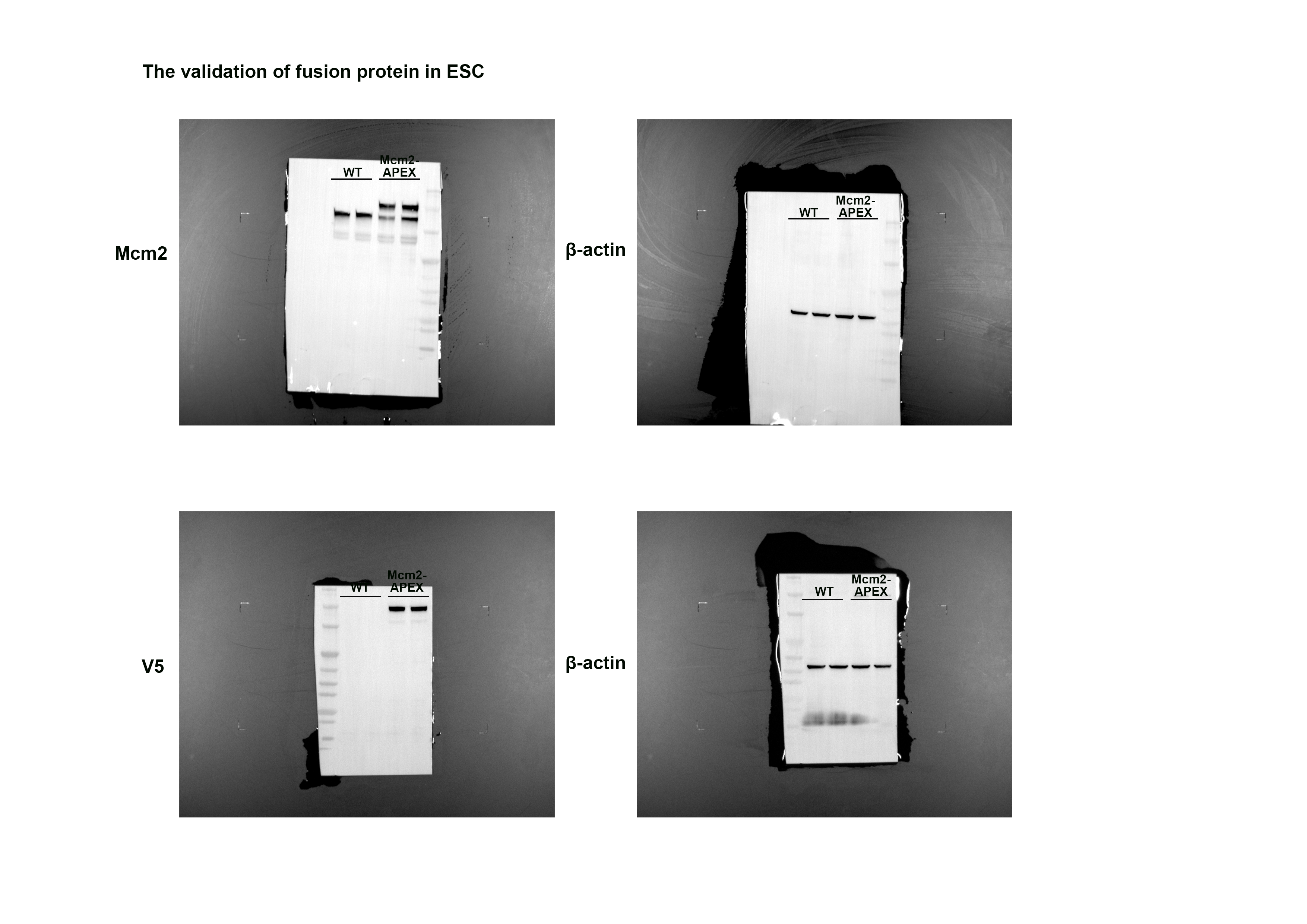

Supplement: Supplementary file 1 [file ijms-26-01020-s001.zip › supplementary figure S1.png]

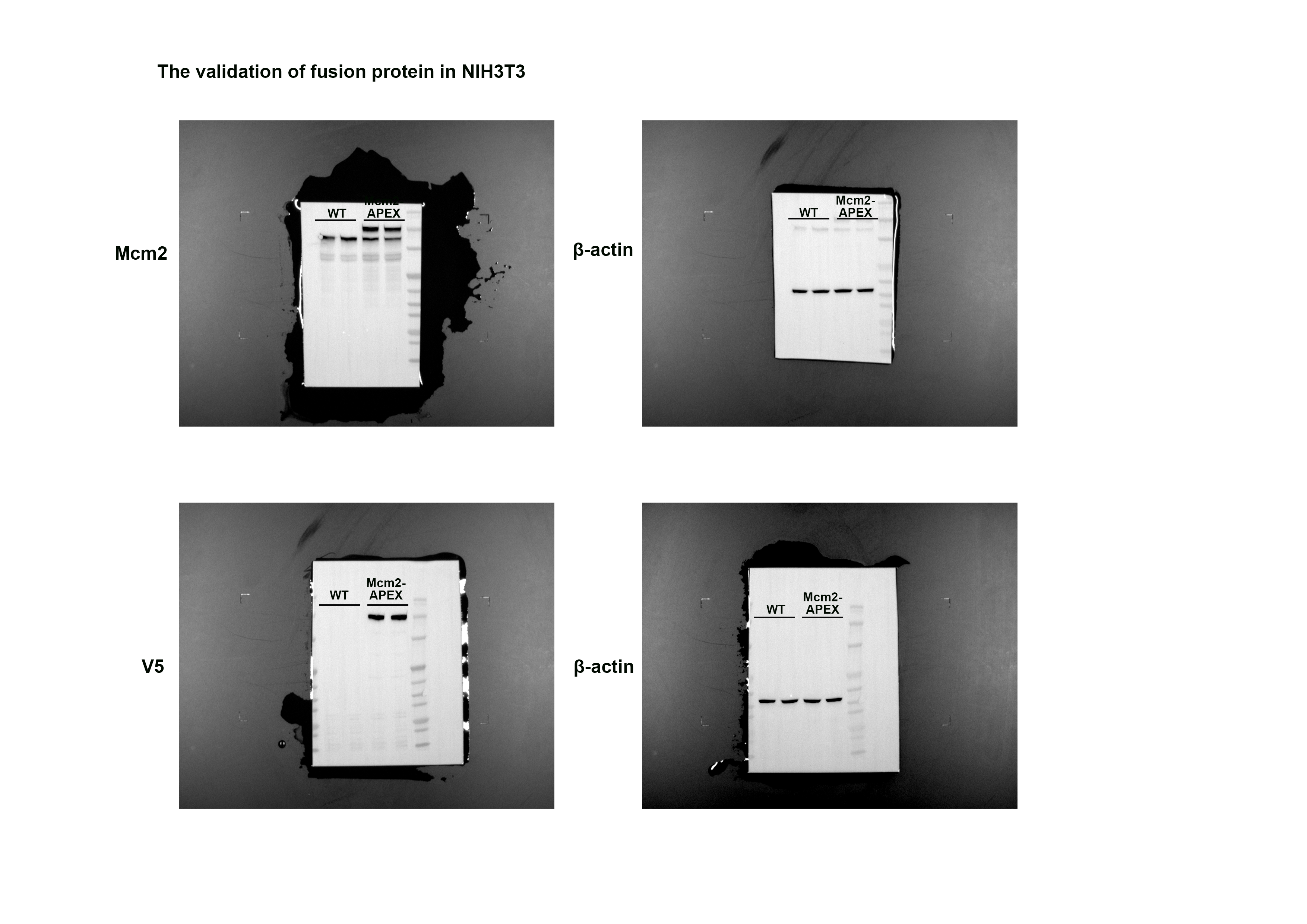

Supplement: Supplementary file 1 [file ijms-26-01020-s001.zip › supplementary figure S2.png]

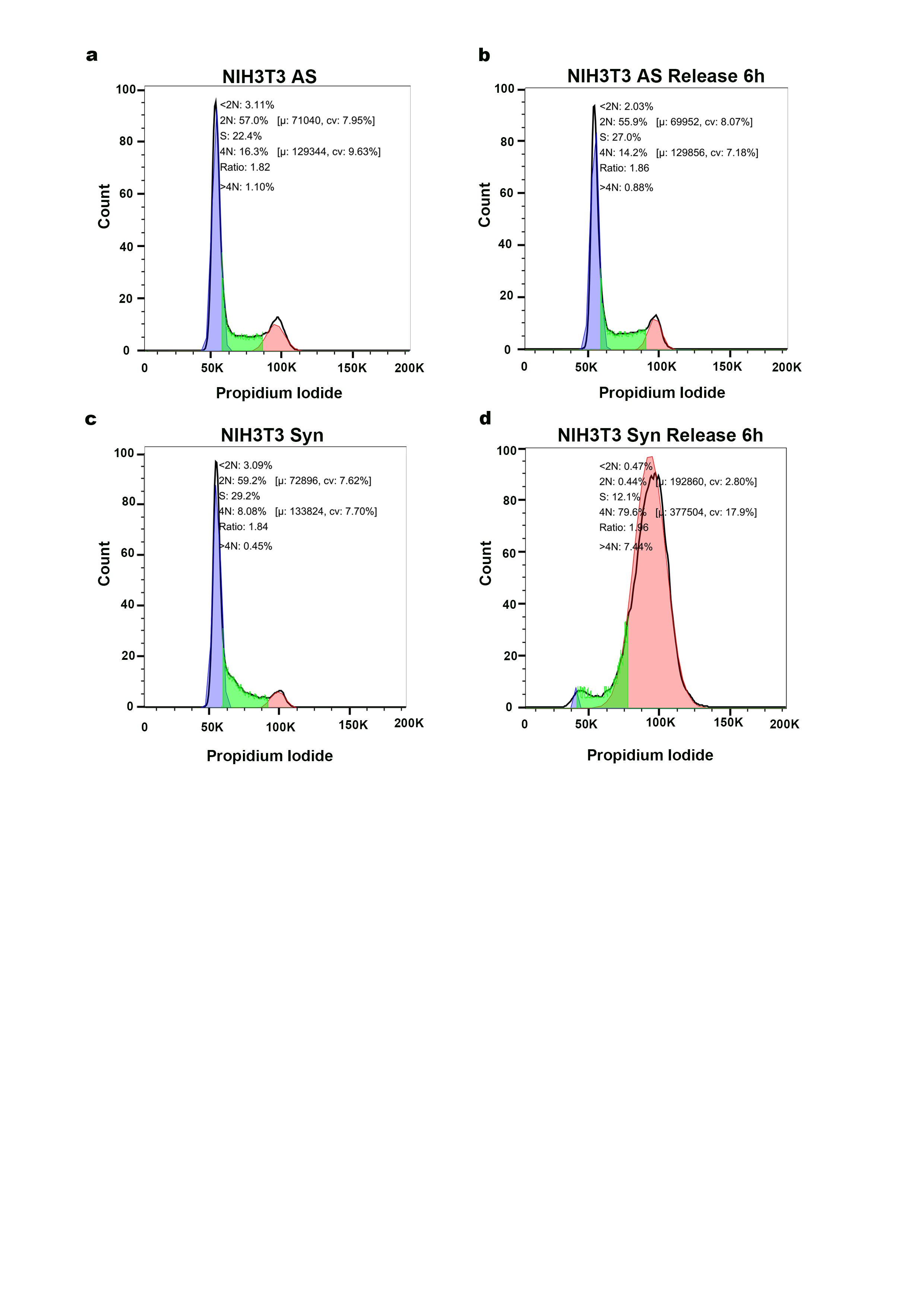

Supplement: Supplementary file 1 [file ijms-26-01020-s001.zip › Supplementary Figure S3.png]

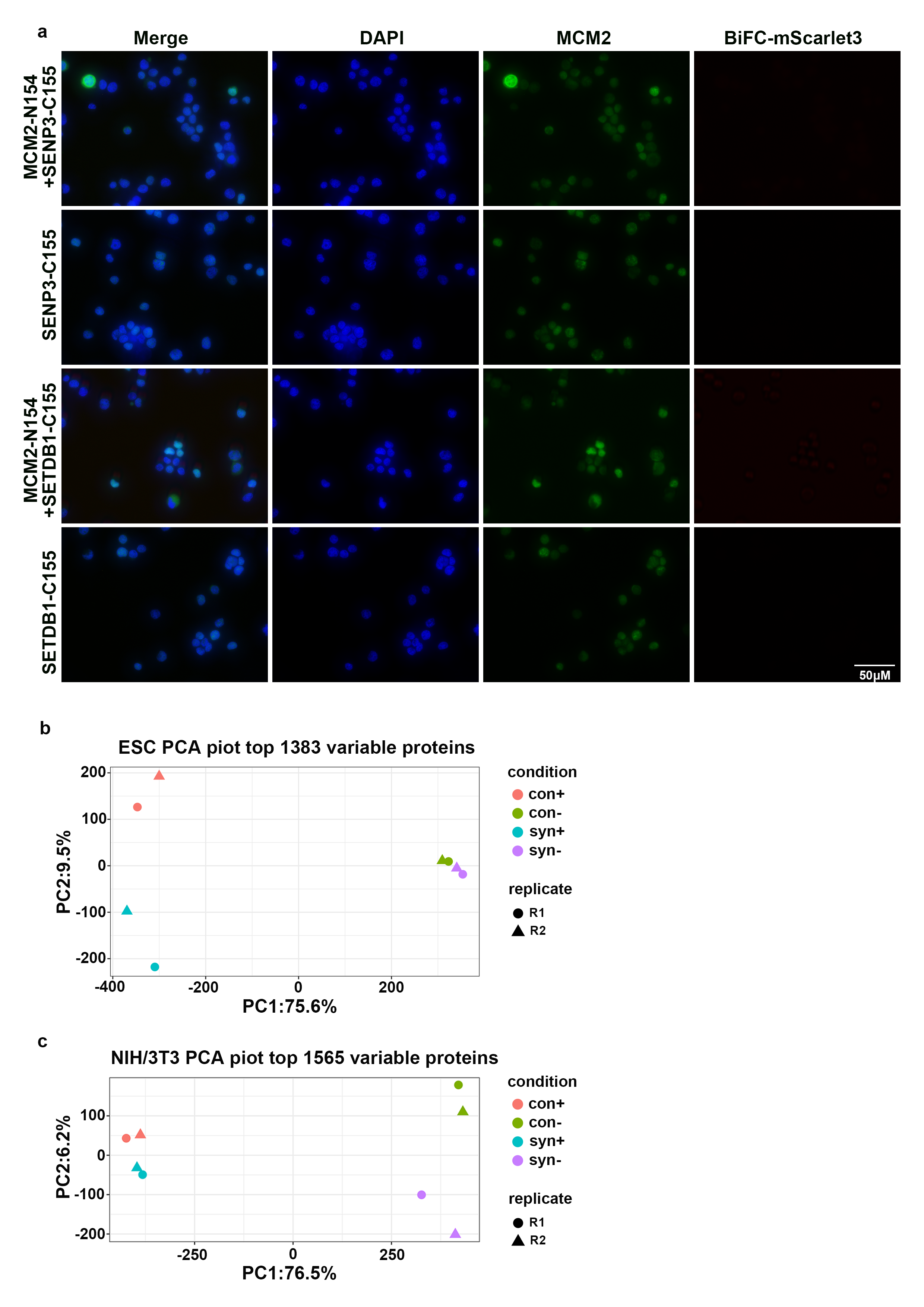

Supplement: Supplementary file 1 [file ijms-26-01020-s001.zip › Supplementary Figure S4.png]

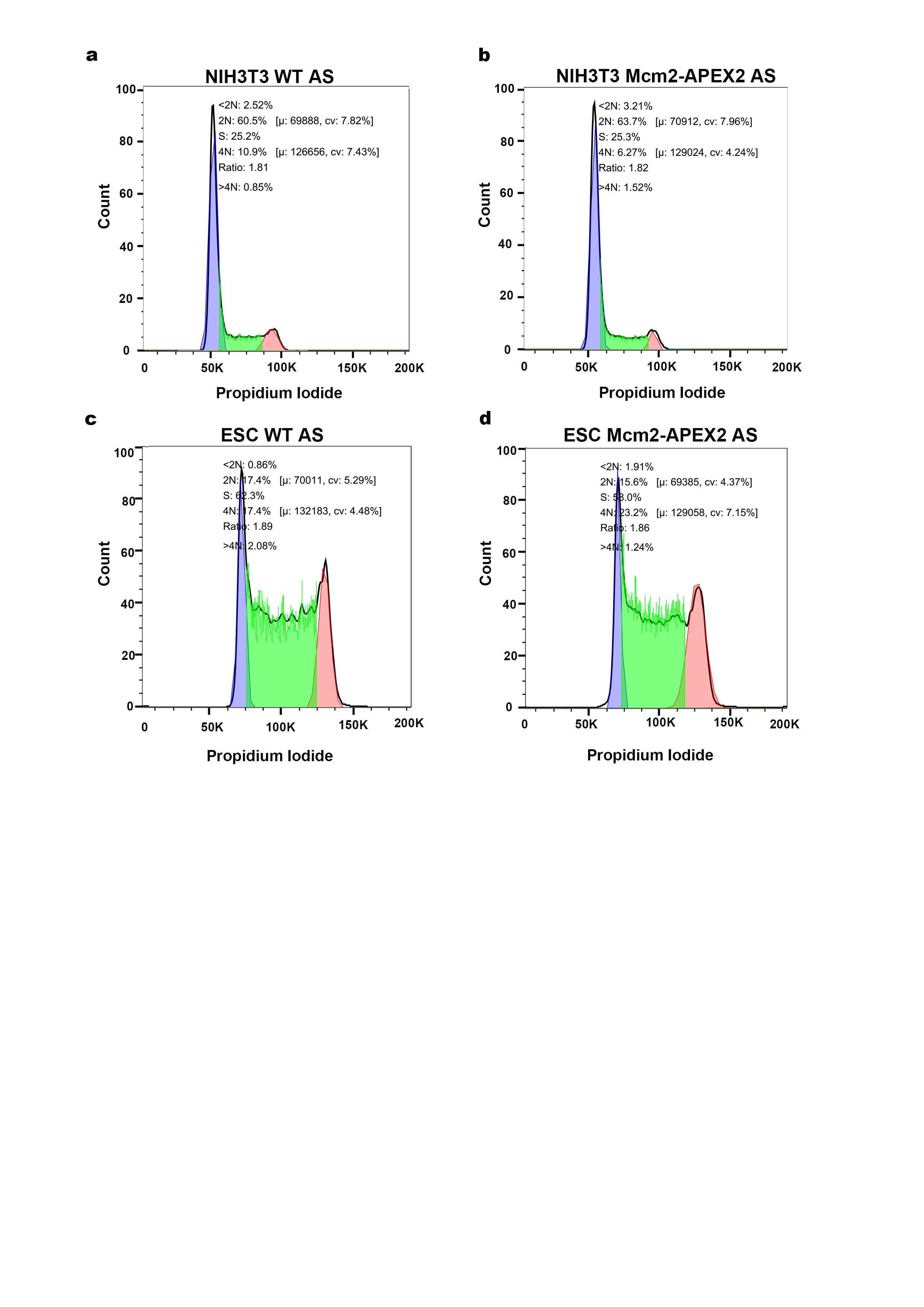

Supplement: Supplementary file 1 [file ijms-26-01020-s001.zip › Supplementary Figure S5.png]
